# Adult-onset status epilepticus in patients with COQ8A coenzyme Q10 deficiency: A case series

**DOI:** 10.1016/j.ebr.2024.100716

**Published:** 2024-10-06

**Authors:** Panagiota-Eleni Tsalouchidou, Clara Juenemann, Wiebke Hahn, Felix Zahnert, Leona Möller, Lukas Hakel, André Kemmling, Katja Menzler, Ole J. Simon, Lars Timmermann, Susanne Knake, Felix Bernhard

**Affiliations:** a Department of Neurology, University Hospital Gießen and Marburg, Marburg, Germany; b Epilepsy Center Hessen, Department of Neurology, Philipps University Marburg, Marburg, Germany; c Second Department of Neurology, Attikon University Hospital, National and Kapodistrian University of Athens, Athens, Greece; d Department of Neuroradiology, Philipps University Marburg, Marburg, Germany; e Department of Psychiatry and Psychotherapy, Philipps-University Marburg, Marburg, Germany

**Keywords:** COQ8A coenzyme Q10 deficiency, Focal status epilepticus, Epileptic encephalopathy, High-dose CoQ10 treatment, Mitochondrial disease

## Abstract

This case series describes the clinical features, diagnostic challenges, treatment approaches, and outcomes of three adult patients with COQ8A-related CoQ10 deficiency presenting with focal status epilepticus, who were effectively treated at the Department of Neurology, Philipps University Marburg, Marburg, Germany. The patients, all from consanguineous families with the first two being siblings, presented with a late onset of the disease, characterized by progressive cerebellar ataxia and epilepsy, with clinical deterioration and focal status epilepticus occurring in adulthood. The first patient exhibited myoclonic status, while the second and third patients presented with bilateral tonic-clonic seizures followed by focal status epilepticus manifesting with cortical blindness. Despite differing semiologies, all patients displayed similar EEG findings with continuous or nearly continuous occipital sharp waves and spikes. MRI findings revealed focal changes in the Diffusion-Weighted Imaging (DWI) and Fluid Attenuated Inversion Recovery (FLAIR) sequences. Significant clinical improvement was observed following treatment with high doses of CoQ10, with the diffusion restriction abnormalities being reversible after therapy. In conclusion, consistent with existing literature, we observed a characteristic EEG pattern that can help identify the disease and facilitate early diagnosis and treatment. The diffusion restriction abnormalities on MRI were reversible after therapy, and high doses of CoQ10 proved beneficial even in cases of status epilepticus.

## Highlights

• COQ8A-related deficiency may present as focal status epilepticus in adulthood*.*
• High-dose CoQ10 improved symptoms in COQ8A-related status epilepticus.
• EEG and MRI findings help early diagnosis of COQ8A-related CoQ10 deficiency.

## 1 Introduction

Coenzyme Q10 (CoQ10), also known as ubiquinone, is a crucial component of the mitochondrial respiratory chain and a fat-soluble element of the electron transport chain, necessary for aerobic cellular respiration, primarily in mitochondria [1]. Deficiencies in CoQ10 can lead to a variety of clinical manifestations, including cerebellar ataxia, epilepsy, encephalopathy, myopathy, macular degeneration, and nephropathy [2], [3].

COQ8A, also known as AarF Domain Containing Kinase 3 (ADCK3), is a gene essential for the biosynthesis of CoQ10. It is inherited in an autosomal recessive manner, with variable clinical manifestations that can also show pathogenicity in heterozygous individuals [4], [5], [6]. Mutations in this gene can cause CoQ10 deficiency, resulting in reduced intracellular concentrations of CoQ10 [7]. This condition can manifest in both adults and children, exhibiting symptoms that overlap with those of other mitochondrial diseases, such as polymerase gamma (POLG)-related disorders [5], [6].

Patients with COQ8A-related CoQ10 deficiency often present with epileptic encephalopathy, cerebellar ataxia, and myopathy. The few cases reported in the literature with electroencephalographic findings show a common electroencephalographic (EEG) pattern with continuous occipital bilateral epileptiform discharges, and some patients experience clinical improvement following high doses of CoQ10 supplementation [5], [6].

Adult-onset focal status epilepticus is a rare but serious manifestation of COQ8A-related CoQ10 deficiency. This case series aims to describe the clinical features, diagnostic challenges, treatment approaches, and outcomes of adult patients with COQ8A-related CoQ10 deficiency presenting with this condition.

## 2 Methods

This case series analyzes the clinical, electroencephalographic, and imaging findings, as well as the treatment approaches, of three patients diagnosed with CoQ10 deficiency who presented with focal status epilepticus. Data were collected retrospectively from patients admitted to the Department of Neurology at the Philipps University Marburg. Patients included in this case series were diagnosed with CoQ10 deficiency before or during the status epilepticus. This was confirmed through genetic analysis revealing COQ8A (ADCK3) mutations. All patients were treated at the Department of Neurology, Philipps University Marburg.

Clinical data were extracted from patients’ medical records, including detailed medical history and demographic information, clinical presentation and symptoms at the time of admission, EEG findings, Magnetic Resonance Imaging (MRI) results, treatment regimens including dosages of CoQ10 and antiseizure medication (ASM), response to treatment, and follow-up outcomes.

The local Ethics Committee of the University of Marburg approved the report (Ethics Commission Phillips University of Marburg; approval number RS24/186). Informed written consent was obtained from all patients before inclusion in this case series.

## 3 Case reports

### 3.1 Case report: Patient 1

A 47-year-old woman with a history of progressive ataxia and epilepsy was admitted to our hospital in July 2020 due to persistent bilateral myoclonic seizures involving both upper and lower extremities for the past week. The first manifestation of the disease occurred at the age of 26 years when the patient experienced a stroke-like episode and status epilepticus. She had previously shown mild cognitive developmental delay, which manifested during juvenile development and necessitated a change to a school for special education at the age of 14. The patient was from a consanguineous family, with her parents being cousins, which increased the suspicion of a genetic disorder. Genetic testing for mitochondrial encephalomyopathy, lactic acidosis, and stroke-like episodes (MELAS) was negative. Since the initial episode, the patient had been treated with carbamazepine, with no change in treatment regimen due to seizure freedom.

**Fig. 1:**
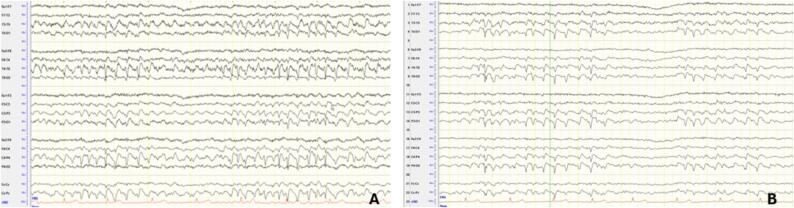
EEG Findings of the first patient: 1A EEG recording during the myoclonic status epilepticus showing continuous posterior bilateral synchronous epileptiform discharges and 1B during the clinical improvement showing a persisting occipital sharp waves pattern. Montage: longitudinal bipolar. Trace duration: 10 s.

On admission, the neurological examination revealed a left-sided predominant cerebellar syndrome characterized by ataxia, slowing of saccades, and scanning speech. The patient's EEG showed generalized slowing with continuous or near-continuous bilateral synchronous occipital and temporo-occipital spikes and sharp waves (Fig. 1A–B). Some myoclonic jerks correlated with the EEG findings, while others did not. As the myoclonus intensified, the electrographic seizures showed spatiotemporal evolution. An MRI revealed cerebellar atrophy, focal diffusion restriction, and FLAIR hyperintensity in the right frontal lobe (Fig. 4A–C). A lumbar puncture showed no inflammation, and neurodegeneration markers and neuronal antibodies were negative.

Despite the escalation of the antiseizure medication through various combinations and high doses of levetiracetam, clonazepam, lacosamide, perampanel, and topiramate, followed by brivaracetam, the patient exhibited continuous clinical deterioration. She was admitted to our intensive care unit, where her myoclonia continued to worsen over more than a month. During this period, the patient developed a respiratory tract infection, which may have contributed to her clinical deterioration by exacerbating the myoclonus and complicating the management of the seizures. As her condition declined further, accompanied by respiratory failure and infection, intubation, propofol anesthesia and tracheostomy were required and maintained for two months without any clinical improvement, despite continuous adjustments to the anti-seizure medication.

Due to the suspicion of mitochondrial disease, a muscle biopsy was performed, which showed no significant abnormalities. Further genetic testing revealed a heterozygous mutation in the COQ8A: c.895C > T(p.Arg299Trp). Subsequently, the patient was treated with high doses of ubiquinone (3000 mg/day). Under this medication, significant improvement was observed with a gradual reduction in the frequency and intensity of the myoclonia. This resulted in the decannulation of the patient one week after the initiation of the treatment with ubiquinone. Despite the clinical improvement, the bioccipital sharp wave pattern persisted at a lower frequency and amplitude (Fig. 1A and B). The MRI findings improved four weeks after the patient's clinical improvement. Due to rehabilitation treatment, the patient recovered completely within 2–3 months after discharge from the intensive care unit of the Department of Neurology.

### 3.2 Case report: Patient 2

The second patient, a 43-year-old woman and sibling of the first patient was admitted to our intensive care unit due to a bilateral tonic-clonic seizure followed by a focal bioccipital status epilepticus, manifested as visual loss and anosognosia (Anton syndrome), along with impaired awareness. One year earlier, after her sister had been diagnosed with COQ8A-related coenzyme Q10 deficiency, this patient had also been diagnosed with the same condition and had been taking 600 mg CoQ10 per day due to similar clinical manifestations, including cerebellar ataxia, retinitis pigmentosa and rare seizures since the age of 18. Despite the progressive worsening of ataxia over the years, the patient had maintained good seizure control on carbamazepine monotherapy. However, due to an increase in seizure frequency without any known trigger one week before admission, her antiseizure medication had been switched from carbamazepine to lacosamide while continuing the previous dose of CoQ10 at 600 mg/ day.

**Fig. 2:**
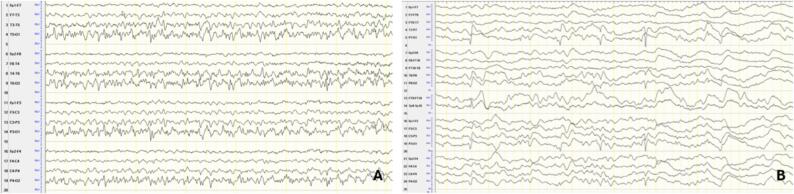
EEG Findings of the second patient: 2A EEG recording during the focal status epilepticus manifesting with cortical blindness and impaired awareness showing almost continuous occipital bilateral synchronous epileptiform discharges and 2B after the clinical improvement showing persisting epileptiform discharges and encephalopathy. Montage: longitudinal bipolar. Trace duration: 10 s.

The patient's EEG showed generalized slowing and continuous bilateral synchronous occipital sharp waves, which exhibited spatiotemporal evolution in the posterior regions. Clinically, this was manifested as visual loss and impaired awareness (Fig. 2). An MRI showed cerebellar atrophy and diffusion restriction with correlation in the ADC in both occipital lobes (Fig. 4D–F).

Initial treatment with high doses of lacosamide failed to control the seizures, so 400 mg of brivaracetam per day was added to control the status epilepticus stiripentol 450 mg/ day and clobazam 15 mg/ day were also added to the anticonvulsive treatment with Brivaracetam. Finally, the CoQ10 dose was increased to 3000 mg/ day. Additionally, the patient was treated with vitamin C, levocarnitine, biotin, and vitamin B1 supplements [8], [9]. With this treatment combination, the patient presented reduced episodes of impaired awareness and gradual improvement of cortical blindness, returning to her baseline state within a week. One week later, the patient regained vision. MRI findings showed improvement two weeks after the clinical improvement and recovery of the patient's vision.

### 3.3 Case report: Patient 3

The third patient, a 27-year-old male from another consanguineous family**,** with his parents being first cousins, was admitted for the first time to our intensive care unit after a series of bilateral tonic-clonic seizures associated with cortical blindness and impaired awareness. The patient had a known history of focal epilepsy, cerebellar atrophy, and developmental delay, including a learning disability and motor coordination deficits. His seizures had begun at the age of 13 and had initially been well controlled. He also had a history of a stroke-like episode at the age of 15. Genetic testing for MELAS, including a muscle biopsy, had been negative. However, over the past year, there had been a notable increase in seizure frequency and severity, resulting in multiple hospitalizations.

**Fig. 3:**
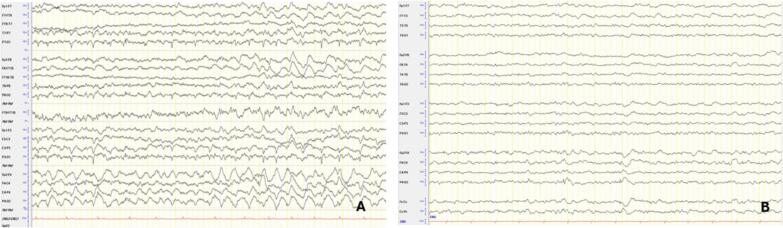
EEG Findings of the third patient: 3A EEG recording during the focal status epilepticus manifesting with impaired awareness showing evolution of the discharges bilaterally and 3B after the clinical improvement showing rare epileptiform discharges and encephalopathy. Montage: longitudinal bipolar. Trace duration: 10 s.

**Fig. 4:**
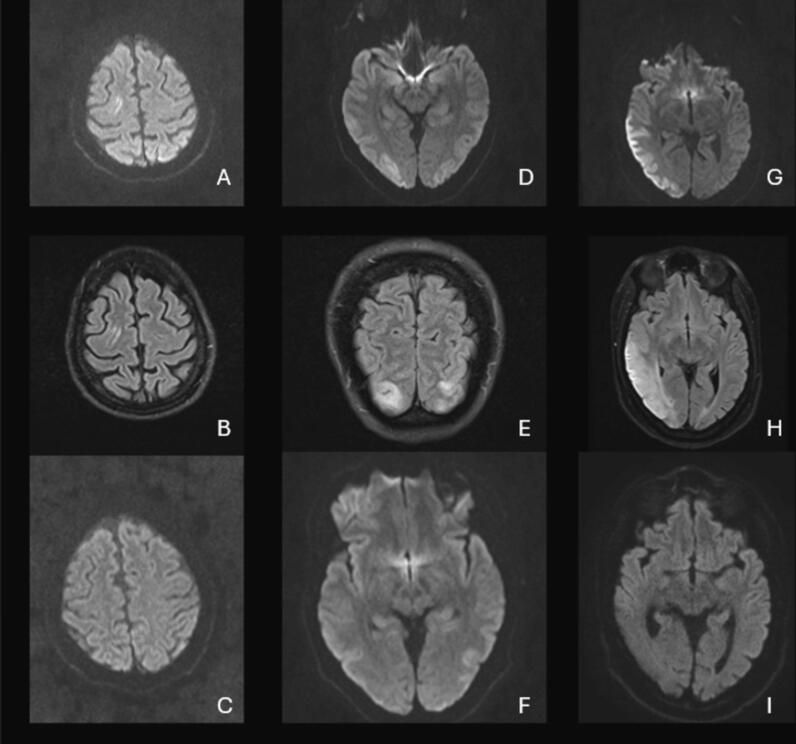
(A) Axial DWI showing diffusion restriction of the right frontal lobe, (B) axial FLAIR showing hyperintensity on the right frontal lobe and (C) axial DWI showing no diffusion restriction four weeks after effective treatment of the first patient with focal status epilepticus and COQ8A-related CoQ10 deficiency presenting with myoclonia. (D) Axial DWI showing bilateral temporooccipital diffusion restriction, (E) coronal FLAIR showing bilateral occipital hyperintensity and (F) axial DWI showing improvement in the diffusion restriction two weeks after clinical improvement of the second patient with COQ8A-related CoQ10 deficiency presenting focal status epilepticus and cortical blindness. (G) Axial DWI showing right temporooccipital diffusion restriction, (H) axial FLAIR showing signal hyperintensity in the same region and (I) axial DWI showing improved findings after treatment of the third patient with COQ8A-related CoQ10 deficiency. *DWI: diffusion-weighted image; FLAIR: Fluid Attenuated Inversion Recovery.

The EEG showed generalized slowing of the background activity, with right occipital and temporal sharp waves and intermittent focal slowing, primarily in the right temporal region. Seizures were captured on EEG during recurrent episodes, which ultimately led to the patient’s admission to the intensive care unit (Fig. 3A–B). An MRI scan during the admission revealed cortical and subcortical edema in the right temporo-occipital region (Fig. 4G–I). These findings were initially attributed to either an encephalitic process or postictal changes. Further imaging showed progressive cortical and subcortical diffusion restriction with partial correlation in ADC abnormalities and edema in these regions, including the right frontal area, indicating ongoing cerebral injury related to seizure activity.

Upon admission, the patient was initially treated with lamotrigine, valproic acid, and clobazam. Due to recurrent seizures, the patient received emergency treatment with levetiracetam (2.5 g i.v.) and midazolam, which temporarily controlled the seizures. Despite this, the seizures persisted, necessitating transfer to the intensive care unit. The patient was then treated with ceftriaxone for suspected infection due to elevated temperature, dexamethasone for edema management found on MRI, and acyclovir, which was later discontinued after negative viral PCR results. Lamotrigine was switched to brivaracetam, valproate was replaced with lacosamide due to the clinical suspicion of potential mitochondrial disease based on the patient's history, and supportive therapy with oral coenzyme Q10 30 mg per day was initially started before genetic confirmation of the suspected mitochondrial disease. With this treatment, the frequency and severity of seizures decreased significantly, and there was a notable improvement in cognitive function and responsiveness. MRI scans showed a reduction in cerebral edema, and the patient's visual and motor functions improved, though he continued to exhibit motor coordination deficits consistent with his chronic condition. After clinical improvement and due to the unconfirmed suspicion of mitochondrial disease, the patient was discharged on CoQ10 at 30 mg/ day and additional antiseizure medication with lacosamide (300 mg/ d), brivaracetam 400 mg/ d, and clobazam 15 mg/ d. Genetic testing was later positive for a homozygous mutation in the COQ8A c.802 T > C(p.Cys268Arg) and confirmed the diagnosis after the patient's clinical improvement.

The patient was admitted urgently for the second time, seven months after his first admission, due to recurrent epileptic seizures with altered semiology compared to previous seizure patterns. On the morning of his admission, the patient experienced focal impaired awareness cognitive seizures, which progressed to clonic jerks in the right arm. Initial emergency treatment included 5 mg midazolam and 200 mg lacosamide. Persistent symptoms led to transfer to the neurological intensive care unit. An EEG confirmed a left-sided status epilepticus, prompting the addition of increased doses of lacosamide, perampanel, and clobazam along with the preexisting brivaracetam. After a CoQ8A mutation with coenzyme Q10 deficiency had been confirmed between the two admissions, the preexisting supplementation of 30 mg CoQ10 per day was directly increased to 4000 mg/day and additional supplements with vitamin C, levocarnitine, biotin, and vitamin B1 were initiated. However, the seizures persisted, and the patient showed metabolic decompensation and lactic acidosis, necessitating endotracheal intubation and sedation with midazolam and esketamine. Following these interventions, the patient was successfully extubated two days later, displaying mild right arm paresis (Todd's paresis) but no aphasia or neglect. Post-treatment EEG showed no status epilepticus. A cranial MRI revealed left frontoparietal and occipital edema corresponding to the initial continuous EEG pattern of the status epilepticus. Two weeks after follow-up MRI scans confirmed a reduction in cerebral edema and cortical and subcortical diffusion restriction.

## 4 Discussion

The patients presented illustrate the clinical complexity and variability of COQ8A-related coenzyme Q10 (CoQ10) deficiency, particularly focusing on the neurological manifestations and treatment responses of focal status epilepticus in three patients with confirmed COQ8A mutations. Despite having the same genetic basis, each patient displayed a combination of common and distinct clinical features, disease progression, electroclinical and imaging findings as well as outcomes after treatment.

All three patients had a consanguineous family history, with the first two being siblings. They presented with a progressive cerebellar syndrome, encephalopathy, myoclonia, visual impairment, a history of stroke-like episodes and epilepsy—a phenotype reported in most cases of COQ8A-related CoQ10 mitochondrial disease [2], [5], [6], [10]. Notably, all patients had a stable disease course following the initial manifestation and experienced an acute clinical deterioration in adulthood with refractory status epilepticus after many years of relative stability, particularly regarding seizure frequency. No definite trigger such as infection was identified for the seizure deterioration and status epilepticus in all three patients. In all cases, although mitochondrial disease was initially suspected due to stroke-like episodes and epilepsy, and more common conditions such as MELAS had been tested negative, a definitive diagnosis was only made after clinical deterioration with status epilepticus prompted more extensive genetic testing to identify the underlying cause. This deterioration led to expanded genetic testing, which confirmed COQ8A primary CoQ10 deficiency. This diagnosis guided alterations in treatment interventions for status epilepticus including high doses of CoQ10 (3000–4000 mg/day), resulting in a gradual and significant clinical improvement in each case.

EEG findings in these patients revealed significant similarities. In the first two patients, electroencephalographic findings demonstrated bilateral synchronous, continuous or fast continuous sharp waves and spikes, predominantly in the occipital and temporo-occipital regions, while the third patient, during the second admission, exhibited a similar pattern, primarily localised to the left side. These findings have been reported in the literature in cases of the same mutation, indicative of a common electroencephalographic pattern in COQ8A-related CoQ10 deficiency [5]. The MRI findings exhibited common features as well. Cerebellar atrophy, focal diffusion restriction, and FLAIR hyperintensity were identified in all patients. The different localisation of the diffusion restriction findings in each patient might reflect a variation in the localisation of the underlying disease process or a result of prolonged seizure activity in these specific brain regions. While focal diffusion restriction is commonly associated with stroke-like episodes in patients with mitochondrial disease and often attributed to exacerbated mitochondrial dysfunction, these lesions might also represent further exacerbated *peri*-ictal MRI abnormalities (PMA), given their association to continuous seizure activity, which further aggravates the underlying condition [11], [12]**.**

## 5 Limitations

This case series highlights the clinical and radiological improvements observed with high-dose CoQ10 supplementation in patients with ADCK3-related CoQ10 deficiency. However, the findings are based on a limited number of cases, and larger studies are needed to validate these observations. Such studies could provide more comprehensive data on the efficacy of CoQ10 supplementation in treating seizures and help establish standardized treatment protocols for this condition, especially in cases of refractory status epilepticus.

## 6 Conclusion

In conclusion, this case series presents three patients with COQ8A-related CoQ10 deficiency and adult-onset status epilepticus, who had previously maintained a stable seizure frequency under medication. Moreover, the recognition of symptoms such as stroke-like episodes, progressive cerebellar ataxia and epilepsy along with the characteristic electroencephalographic pattern of continuous bilateral occipital sharp waves can contribute to the early diagnosis of COQ8A-related CoQ10 deficiency. Treatment with high doses of CoQ10 has been effective in improving symptoms of status epilepticus in these patients. Thus, suspecting and differentiating COQ8A-related disease from other mitochondrial disorders can be significantly beneficial. This case series highlights the importance of tailored treatment strategies, emphasizing the role of genetic testing and high dosages of CoQ10 supplementation in managing severe epilepsy and status epilepticus in patients with COQ8A-related CoQ10 deficiency.

## 7 Consent for publication

All the authors consent to the publication of this manuscript in Epilepsy and Behavior Reports.

## Ethical statement

We confirm that we have read the Journal’s position on issues concerning ethical publication and affirm that this report aligns with the provided guidelines.

## CRediT authorship contribution statement

**Panagiota-Eleni Tsalouchidou:** Writing – original draft, Methodology, Formal analysis, Conceptualization. **Clara Juenemann:** Data curation. **Wiebke Hahn:** Writing – review & editing, Data curation. **Felix Zahnert:** Data curation. **Leona Möller:** Writing – review & editing, Data curation. **Lukas Hakel:** Data curation. **André Kemmling:** Data curation. **Katja Menzler:** Writing – review & editing, Data curation. **Ole J. Simon:** Writing – review & editing, Data curation. **Lars Timmermann:** Data curation. **Susanne Knake:** Writing – review & editing, Data curation. **Felix Bernhard:** Data curation.

## Declaration of competing interest

The authors declare that they have no known competing financial interests or personal relationships that could have appeared to influence the work reported in this paper.
